# Wavelet-Based Tremor Quantification From Wrist-Worn Sensor Data in Home-Dwelling People With Parkinson’s Disease

**DOI:** 10.1109/JTEHM.2025.3648704

**Published:** 2025-12-25

**Authors:** Haakon Reithe, Monica Patrascu, Juan C. Torrado, Elise Førsund, Bettina S. Husebo, Simon U. Kverneng, Erika Sheard, Charalampos Tzoulis, Brice Marty

**Affiliations:** Centre for Elderly and Nursing Home MedicineDepartment of Global Public Health and Primary CareUniversity of Bergen 1658 5007 Bergen Norway; Neuro-SysMed CenterUniversity of Bergen 1658 5007 Bergen Norway; Norwegian Computing Centre 0373 Oslo Norway; Neuro-SysMed CenterHaukeland University Hospital 60498 5009 Bergen Norway; K. G. Jebsen Center for Translational Research in Parkinson’s DiseaseUniversity of Bergen 1658 5007 Bergen Norway; Department of Clinical MedicineUniversity of Bergen 1658 5007 Bergen Norway

**Keywords:** Parkinson’s disease, tremor quantification, multi-resolution analysis, wearable devices, free-living conditions

## Abstract

Objective: Tremor symptoms in Parkinson’s disease (PD) are challenging to assess due to low resolution and subjectivity from standard clinical scales. To address this, wearable devices have been used, but algorithms have been relying on controlled or limited activity conditions. Our objective is to create a context-independent metric quantifying tremor in free-living conditions to bridge the gap between biomedical engineering and the PD field. Methods and Procedures: We designed an algorithm which computes a tremor index (TI) from accelerometer data, collected via the Empatica E4 worn on the wrist by home dwelling people with PD. For validation, we use a within-participant design, comparing the TIs of the most and least tremor-affected hand. We included seven participants with unilateral tremor, monitored for two weeks each. The algorithm is able to compute TIs for a set of frequencies identified in literature as associated with different tremor types (3–12 Hz), over adjustable sampling time windows. Results: We show that the most tremor-affected hand yields a higher TI than the other hand for frequency sets that are individual to each person, in particular around 5-6 Hz where rest tremor typically occurs. We find that we can disambiguate tremor across 3-12 Hz from general movement and resting states. The number of frequencies with inter-hand separation correlate with the MDS-UPDRS part III tremor items. Conclusion: The designed tremor quantification algorithm can quantify tremor symptoms over time for people with PD and can be used to identify the individualized frequency ranges where these movements happen, in free-living conditions.

Clinical and Translational Impact: By utilizing pervasive, easy-to-use wrist-wearable devices equipped with accelerometers, the proposed index has the potential to change how clinicians and researchers track tremor symptoms in the daily lives of patients.

## I. Introduction

Parkinson’s disease (PD) is the most common neurodegenerative movement disorder [1]. It involves loss of dopaminergic neurons in the substantia nigra pars compacta, resulting in bradykinesia combined with rest tremor (RT) and/or rigidity, collectively termed *motor parkinsonism* [2]. RT occurs in more than half of people with PD [3], and is characterized by tremor in a body part that is inactive and supported against gravity [4]. RT diminishes or is suppressed during goal directed movement, but may re-emerge during activity such as walking or maintaining a posture [4], as well as increase during mental or emotional stress [5], [6]. In addition to RT, action tremor (AT) also commonly occurs in PD [7] and is characterized by tremor occurring during deliberate limb movement, such as when reaching for an object (kinetic tremor) or when maintaining a position against gravity (postural tremor) [4].

Motor parkinsonism is the core feature of current clinical diagnostic criteria for PD [8], underscoring the importance of accurate motor assessment in both clinical settings and research. The current gold-standard for motor assessment in PD is the Movement Disorders Unified Parkinson’s Disease Rating Scale (MDS-UPDRS) [9], which, while widely used and recognized, is still limited due to its subjective nature and coarse resolution [10].

In the past decade, a growing body of research has been exploring the use of wearable sensor technology for quantifying tremor in a more objective manner [11]. Several approaches have been proposed, such as the use of accelerometers in controlled laboratory settings to detect tremor [12], [13], [14] or mobile applications that prompt the participant to complete a battery of tasks and use the sensors of the smart phone to measure tremor, among other symptoms [15]. Although these approaches highlight different solutions to more objective measures of tremor in PD, lab-based methods are time consuming and labor intensive, and mobile applications require digital competence which some older adults with PD may not possess. Therefore, shifting the focus to unobtrusive continuous monitoring may potentially provide deeper insight into symptoms over time [10].

Research investigating tremor detection and quantification in free-living settings has utilized complex machine learning algorithms using several parameters [16], [17], [18] or sensors [19] to estimate the occurrence of tremor. Braybrook et al. created a time metric of tremor, termed “percentage of time that tremor was present” [17]. Pope et al. detected tremor based on a heuristic threshold classifier [16], McNames et al. developed a two-stage algorithm to discriminate tremor from other activities [18], and Adams et al. developed a threshold-based algorithm for the proportion of tremor over a day [19]. While these works have demonstrated potential for quantification of tremor, they have some important limitations. Previous approaches have focused on detecting instances of tremor typically using algorithms relying on certain contexts such as sitting or standing, which is either determined natively by the algorithm [16], [19], by a secondary algorithm [18], or by omitting parts of the day to contextualize recordings [17]. This may be due to the difficulty of making sense of sensor data without context in unsupervised environments. Recording and analyzing the entire 24 hours may yield a more complete understanding of the tremor condition of the patient. Furthermore, quantifying fluctuations in severity, duration and type of tremor over time could provide valuable information to the treating neurologist for assessing treatment response and adjusting treatments. Thus, there is a need for a context-independent algorithm that enables parameter customization for individuals or groups, allowing for quantification of tremor in real-world scenarios.

In this study, we aim to quantify tremor derived from accelerometer data collected using a wrist-worn wearable sensor in free-living conditions. We hypothesize that multiresolution analysis based on Daubechies wavelets can disambiguate tremor-related movement at different frequencies. To this end, we develop an algorithm that quantifies tremor over time, and validate the algorithm by comparing the most tremor-affected hand with the least affected hand in persons with PD.

## II. Methodology

### A. Study Overview and Design

The current work includes data from 7 participants with PD selected from ActiveAgeing [20], a mixed method cyclical study exploring the use of digital phenotyping as a tool to improve the lives of people with PD, without PD, and informal caregivers. ActiveAgeing consists of two branches, where the DIGI.PARK branch investigates PD in participants recruited from the STRAT-PARK ( $N = 13$) [21] and NOPARK ( $N = 2$) [22] cohorts. Inclusion criteria for the current work were a diagnosis of ‘established’ or ‘probable’ PD, according to the Movement Disorders Society Clinical Diagnostic Criteria for PD [8] and self-reported lateralization of tremor in the upper extremities, where one side exhibited more frequent tremor than the other. A detailed description of the ActiveAgeing protocol is available [20].

In the current work, three types of data were included: a standard clinical scale, self-reported symptoms, and movement measurements derived from a wrist-worn device. The clinical data includes [23]: total score of MDS-UPDRS part III (clinician-assessed motor examination, range 0 – 132: ≤ 32 mild, ≥ 59 severe), single tremor items from the scale (0: no symptom, 1 – 4: light to severe), and the Hoehn and Yahr stage (severity of PD dysfunction, 0: asymptomatic, 1 – 5: ‘unilateral symptom involvement only’ to ‘wheelchair user or bedridden unless aided’). For the self-reported symptoms, participants were asked what motor symptoms they experienced and whether the symptoms had a unilateral profile. Movement data was collected using the Empatica E4, a multi-sensor device with several sensors, including a three-axis accelerometer with a sampling rate of 32 Hz [24].

**FIGURE 1.**
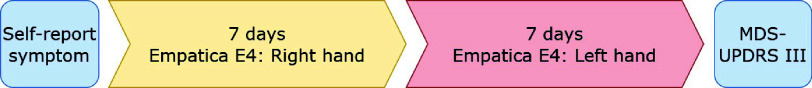
Data collection timeline. MDS-UPDRS: Movement Disorders unified parkinson’s disease rating scale.

Data was collected over two weeks. Self-reported symptom data was collected at baseline. Participants wore the Empatica E4 device for approximately 7 days on one wrist and 7 days on the other (Fig. 1). Data from devices were retrieved every 48 hours to accommodate the battery capacity of Empatica E4. To avoid data gaps during recharging, alternating devices were used, as those with depleted battery were swapped with fully-charged, ready-to-record ones. The clinical scale data were collected at the end of the period. A comprehensive description of the protocol is detailed in our previous work, including challenges and lessons learned [25].

*Ethics.* The STRAT-PARK (74985) and NOPARK (2017/2083) studies are both approved by the Regional Ethics Committee. The DIGI.PARK branch has also received approval from the Norwegian Centre for Research Data (NSD-792472). The study is conducted in accordance with the Declaration of Helsinki: Ethical Principles for Medical Research Involving Human Subjects and adheres to the EU General Data Protection Regulation 2016/679 (GDPR). The study participants provided their written informed consent to participate and were informed of their right to withdraw from the study at any time, without prejudice to their future treatment and care.

### B. Tremor Quantification Algorithm Development

In this article, we develop a tremor quantification algorithm that is independent of learning from data. To this effect, we employ signal processing techniques to obtain an individualized assessment of tremor over a wide frequency range. The algorithm has both clinical and research uses, in two modes: **I.** identifying individual frequency ranges and **II.** tracking tremor over time.

**Concept. Multi-resolution analysis (MRA)** is a signal processing technique that untangles the harmonics of a periodic signals or time series [26], [27], [28], [29]. This method is based on wavelet decomposition, which transforms a signal by displacing and dilating itself, with the purpose of detecting and analyzing various properties of the signal. Here, we choose Daubechies wavelets for their property of preserving the energy of the signal [30]. The MRA result is a set of component signals (i.e., levels) that describe fluctuations at different frequencies around a base evolution (i.e., the smooth). In this article, we utilize the levels to describe ‘smaller movements’ around the smooth, which represents the ‘larger motion’ of the arm in the 3-D space. Relative to each other, the former are quick-varying with lower amplitudes (e.g., tremor), while the latter are slow-varying with either larger amplitudes (e.g., daily activities) or stationary state (e.g., limb at rest, supported against gravity).

**Signal selection.** Tremor quantification in free-living conditions is a challenging problem due to the contextual covariates that affect how tremor reflects in sensor data. In clinical settings, the MDS-UPDRS III is designed to optimize tremor observability. In daily life, actions and motion that are part of regular activities can overwhelm the way smaller movements related to tremor appear in the digital data. This is known as a degree-of-freedom problem: we cannot measure more variables than available sensors. With a single smartwatch equipped with a 3-axis accelerometer we will not be able to disambiguate symptoms from activities. Therefore, choosing the signal that can best reflect tremor symptoms in the presence of regular activities is paramount.

**FIGURE 2.**
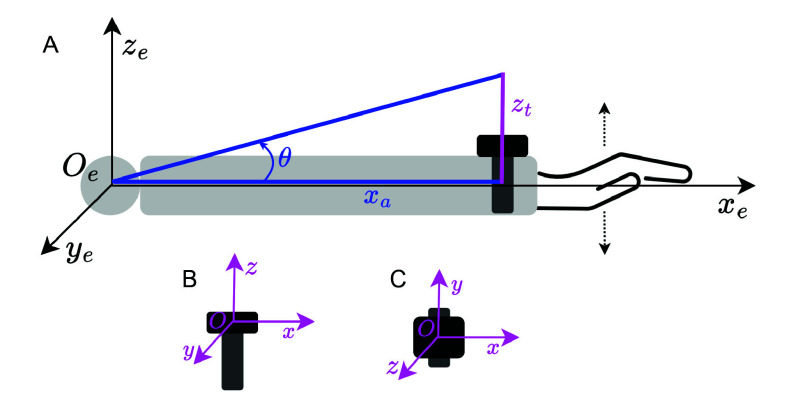
Selecting the relevant signal for tremor quantification based on arm kinematics and typical daily movement. A. Coordinate system of arm kinematics; B. Empatica E4 side view; C. Empatica E4 top view.

Based on wrist/hand motion trajectories during tremor, we exclude the $Ox$ axis movement of Empatica E4 (Fig. 2): forward motion parallel to the fingers. Based on wrist/hand action ubiquity during regular activities, we exclude the $Oy$ axis movement of Empatica E4 (Fig. 2), which is in the same plane as the palm and can be observed as arm swing during walking, using a computer mouse, cleaning surfaces, etc.

The $Oz$ axis of Empatica E4 (Fig. 2) captures movement perpendicular to the wrist/palm plane. The muscle contractions of flexion and extension are detected by the accelerometer on this axis, while rolling motion that drives the device in an elliptical trajectory will also have an $Oz$ component that is detected. This signal is thus likely to contain tremor information while being the least affected by daily activities. The challenge is posed by pronation and supination, which cause the watch change orientation on its $Oz$ axis; this would be difficult to assess without a fixed reference point.

We thus analyze the kinematics of the arm with the fixed origin in the elbow ( $O_{e}$) in the $x_{e}y_{e}z_{e}$ space. Angle $\theta $ is the elevation (spherical coordinates physics convention); independently of lateral movement on the $Oy$ axis, $\theta = \arctan (z_{t} / x_{a})$, where $z_{t}$ is the wrist movement amplitude relative to the elbow and $x_{a}$ is the forearm length (Fig. 2). Angle $\theta $ contains information on flexion, extension, and a direction of rolling. By anchoring the origin $O_{e}$ at the elbow, pronation and supination will be detected as the device flip happens with a variation of $\theta $ dependent on the depth of the wrist. Because the forearm length does not change over time for each person and because the wristband is not displaced along the arm (for proper mounting with snug fit), we fix $x_{a} = 1$ as the neutral operand for division. Thus, we remove the contribution of forward and backward actions performed with the whole arm in the $x_{e}O_{e}y_{e}$ plane. This choice standardizes the elevation calculation across all study participants, as we are not aiming to compare symptoms between participants, but to obtain an individualized estimation of tremor quantity over time. In what follows, we choose $\theta $ as the basis for tremor quantification.

While the motion perpendicular to the wrist/palm plane $x_{e}O_{e}y_{e}$ does not perfectly describe the entire range of tremor kinematics, movements that appear in both RT and AT are observable through angle $\theta $, making this signal an optimal candidate (in these conditions) for tracking tremor in the presence of artefacts introduced by daily activities.

**Tremor frequency range in Parkinson’s disease.** To quantify a general tremor occurrence, we chose a frequency range of 3-12 Hz to ensure that we capture a wide spectrum of different tremor [31] and individual differences in people with PD. Tremor types such as RT and AT have been shown to occur at different frequencies [32], [33], [34], [35] and the occurrence of the different types of tremor varies among people with PD [36].

The Daubechies wavelet levels and smooth are calculated at a frequency set that divides the original signal’s by consecutive powers of two. To extract the movement levels at $f \in \{3, 4, \ldots , 12\}$ Hz, we resample the original acceleration signal from 32 Hz to the nearest sampling frequency that will disambiguate each of the instances of *f*.

**The sampling time (ST)** refers to the period for which the MRA is applied in order to obtain individualized assessments of tremor over the course of days, weeks, etc. In this paper, we choose the ST based on the reported medication onset of action (30-45 minutes), reported medication times of on-the-hour or half-past-the-hour intervals, the Nyquist-Shannon theorem [37], and the principles of discretizing continuous systems [37]: thus, the ST should be a divisor of the medication reaction time by at least a factor of 2. For an average reaction time to treatment between 30-45 minutes, we can choose a ST of 15 minutes. The ST remains a parameter that can be adjusted as needed in different studies or populations.

**The tremor index (TI)** represents the amount of movement at each frequency $f \in \{3, 4, \ldots , 12\}$ Hz. We calculate the TI as the wavelet level’s energy per ST window:

\begin{equation*} TI_{f} = \beta \sum _{i=1}^{q} |\theta _{f}(i)^{2}|, \tag {1}\end{equation*}

where *q* is the size of the ST (15-minute) window in number of datapoints at each frequency *f*, and $\theta _{f}$ is the level for frequency *f*. The scaling operator $\beta $ scales the energy of the level to a measurement unit in the range of milliradians squared per ST window; $\beta $ remains adjustable to other populations or studies. For brevity, we introduce the tremor index unit notation: 1 tiu = 100 mrad^2^/ST.

### C. Use Cases

From a clinical perspective, the TI can allow clinicians to evaluate the quantity of tremor that a patient is experiencing, and how this quantity may change as result of disease progression or medication adjustments. This also applies to research, where quantification of tremor such as the TI may provide a sensitive outcome measure in intervention studies.

#### 1) Mode I. Identifying Individual Frequency Ranges

To identify the individual frequency ranges for tremor occurrence, the patient would be given a smartwatch equipped with a 3-axis accelerometer to be worn for two weeks, switching sides at the midpoint. The distribution of all TI values calculated at ST intervals would then be calculated for each hand. Patients with unilateral tremor will show differences in the side with tremor compared with the other side, and larger separation between the hands for the frequency levels where tremor occurs. Patients with tremor on both sides would show similar TI distributions for the two hands.

#### 2) Mode II. Tracking Tremor Progression Over Time

The proposed algorithm enables tremor tracking over time in free-living conditions. For this, the patient would be given a smartwatch equipped with a 3-axis accelerometer to be worn for as long as the neurologist considers adequate. The TI values calculated at ST intervals would be inspected visually in relation to medication times or other events. The TI values allow for further analysis, such as before-after medication effect, detection of ON/OFF states, etc.

### D. Data Analysis

We prepare the $Oz$ axis acceleration data $z_{t}$ by standardizing the timestamps to Unix time and dividing the data into 24-hour segments (00:00:00 – 23:59:59). Incomplete segments are excluded when at least 50% of the total number of samples expected in daytime data are missing. The daytime period is standardized to 07:01-23:59 based on the self-reported typical sleep-wake cycles by participants. Nighttime is included in the analysis to obtain a 24-hour overview of the quantified TI by fully utilizing the recording capabilities of the wearable device and potentially uncover waking bouts during the night when participants might have experienced tremor.

**FIGURE 3.**
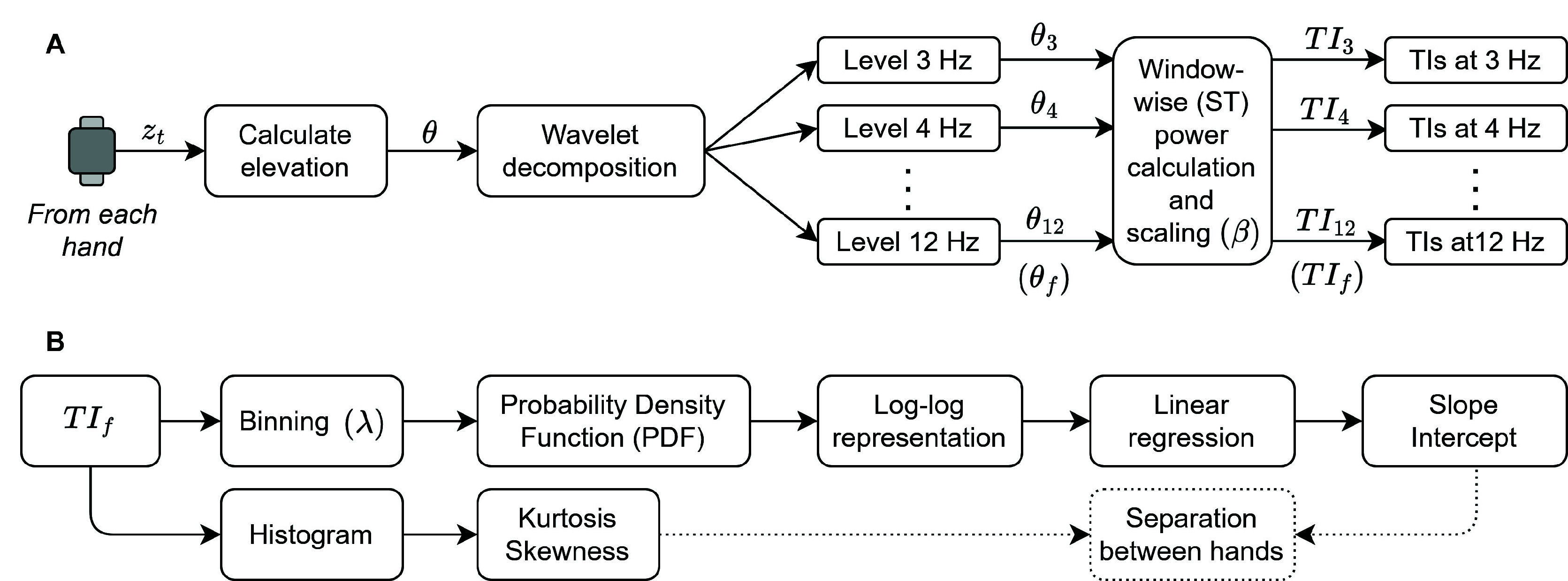
Data analyses pipelines. A. Tremor index (TI) quantification algorithm; B. Hand separation analysis.

Fig. 3 shows the data analyses pipelines. The TI quantification algorithm applies to Modes I and II usage. The hand separation analysis is to determine whether the estimated TI is higher in the most tremor-affected side than the least tremor-affected side and thus test our hypothesis. The upper arm of this pipeline applies to Mode II.

After applying the proposed algorithm on all segments, we comparatively analyze the most tremor-affected hand and the hand least affected by tremor. We perform the comparison for the group, as well as for each participant, to account for individual differences considering the heterogeneity of the disease and the differences in free-living conditions between individuals that may impact the sensor measurements.

To evaluate the differences (separation) between the two hands, we calculate the probability density function (PDF) for each hand (individual as well as all frequencies) based on a binning procedure with bin size $\lambda $. We choose $\lambda $ based on long-tailed distribution linearization. In log-log scale, we apply linear regression (least squares fit) and analyze the differences between the two hands in terms of model slopes and intercepts; for visualization, we utilize a mono-dimensional view of the long-tailed distributions of TIs for each participant, frequency, and hand. In parallel, we complement the analysis by extracting the kurtosis and skewness of the TI distributions for each hand (individual as well as all frequencies) [38].

To investigate the relationship between the inter-hand separation and clinical scores, we quantify the distance between the PDFs in log-log scale along the dimension of the second highest bin, for each participant and each frequency: $D_{f} = log(bin_{f}^{\text {T}}) - log(bin_{f}^{\text {nT}})$ (where T denotes the most tremor-affected hand, and nT the least tremor-affected hand). We consider the distributions separated for at least 30% of maximum TI at that frequency and count them as $C = \sum _{f=1}^{12} c_{f}$, where $c_{f} = 1$ for $D_{f} \geq 0.3\max (TI_{f})$ and 0 otherwise. From the clinical scores, we estimate the ‘tremor symptom load’ as the number of present tremor types weighted by their cumulative intensity: $L = \sum _{k} \text {Type}_{k} \sum _{k} \text {Int}_{k}$, where $k \in \{\text {postural}, \text {kinetic}, \text {rest} \}$ tremor. We then perform a correlation analysis between *C* and *L* normalized to a range of 0 – 10. For verification, we calculate the 95% confidence interval (CI) and apply a paired-samples t-test of the $C-L$ differences.

Summary statistics include individual total MDS-UPDRS III scores, Hoehn and Yahr score, individual tremor scores from the MDS-UPDRS (for each side), medication times before the MDS-UPDRS III examination, and side of tremor occurrence in relation to hand dominance. We also extract the mean and standard deviations of all MDS-UPDRS III scores and total score for the whole group in the tremor items.

All identification, analyses, and visualizations are obtained using Matlab R2023b.

## III. Results

The selected participants had a mean MDS-UPDRS III of 22.4 and a mean Hoehn and Yahr of 1.7. On individual items, rest tremor had the highest score, with postural and kinetic showing the lowest scores. Most participants had unilateral tremor in the non-dominant hand, and medication times at MDS-UPDRS assessment varied (Table 1).

**TABLE 1:** Clinical Characteristics and Symptom Load Summaries

Study participant	MDS-UPDRS III	Hoehn and Yahr	Postural tremor		Kinetic tremor		Resting tremor		Cns	Med	TS	Symptom summaries	
			T	nT	T	nT	T	nT				L	C
P1	30	2	1	0	2	1	1	0	3	15	ND	12	10
P2	27	2	1	0	1	0	1	0	2	360	ND	9	8
P3	34	2	0	0	0	0	3	0	4	60	ND	3	4
P4	11	1	2	0	0	0	2	0	1	60	ND	8	9
P5	29	2	1	0	0	0	3	0	3	120	D	8	6
P6	14	2	0	0	0	0	0	0	0	80	ND	0	4
P7	12	1	0	0	0	0	1	0	1	240	D	1	0
Mean (std)	22.4 (8.9)	1.7 (0.45)	0.71 (0.7)	0	0.42 (0.7)	0.14 (0.3)	1.5 (1.0)	0	2 (1.3)				

T = most tremor-affected hand; nT = least tremor-affected hand; Cns = tremor consistency;

Med = medication before assessment in minutes; TS = Tremor side; ND/D = non-dominant/dominant hand;

L = tremor symptom load; C = number of separated TI frequencies; std = standard deviation.

After preprocessing, a total of 65 24-hour segments were obtained over all participants, with 30 segments (mean 4.5, std. 2) for the least tremor-affected hand and 35 segments (mean 4.8, std. 1.5) for the most tremor-affected hand.

**FIGURE 4.**
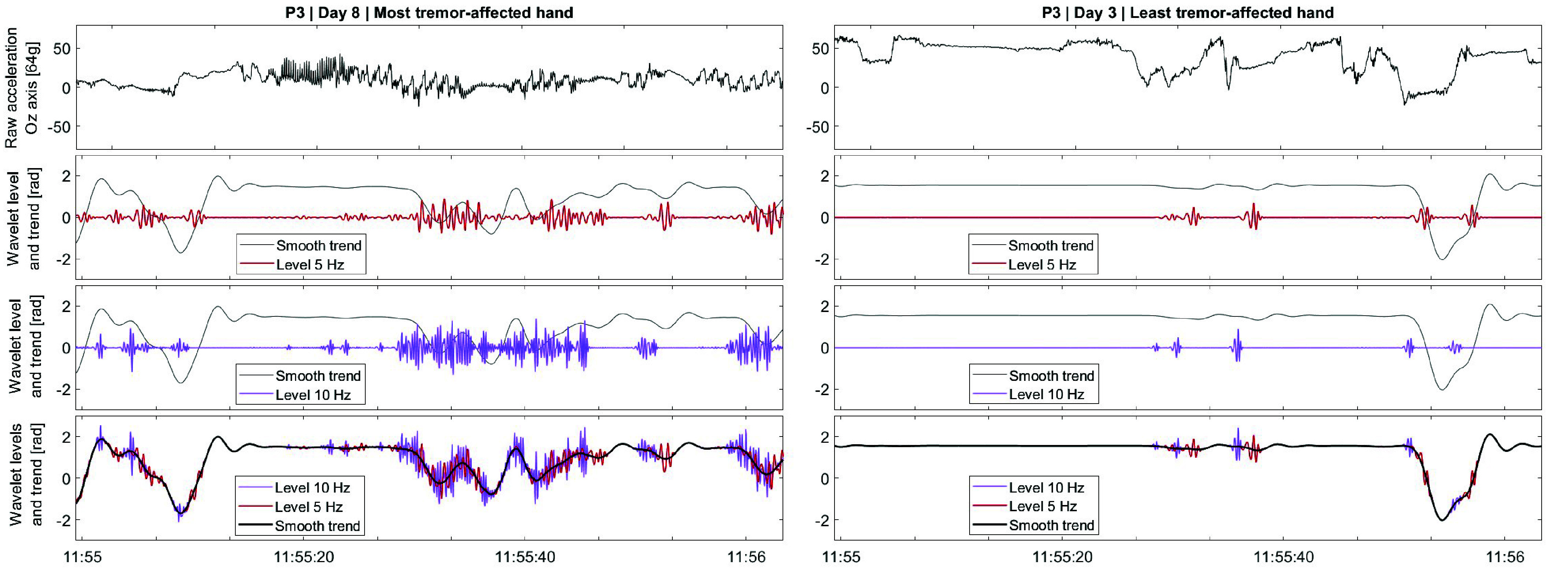
Exemplification of the 5 & 10 Hz wavelet levels with the smooth for participant P3 over 1 minute, while performing the same activity (desk work), with self-reported experienced tremor on Day 8. (Larger version in Supplementary material.)

An example of the wavelet disambiguation for levels 5 & 10 Hz is illustrated in Fig. 4. The 1-minute period shows the TI of both hands during the same activity (desk work) for participant P3 with self-reported tremor experienced in the most-affected hand. From the raw $Oz$ axis acceleration, the algorithm extracts the smaller movement, around the slow-varying smooth trend. An example of a 24-hour segment for both hands and all frequencies is provided in the Supplementary material.

**FIGURE 5.**
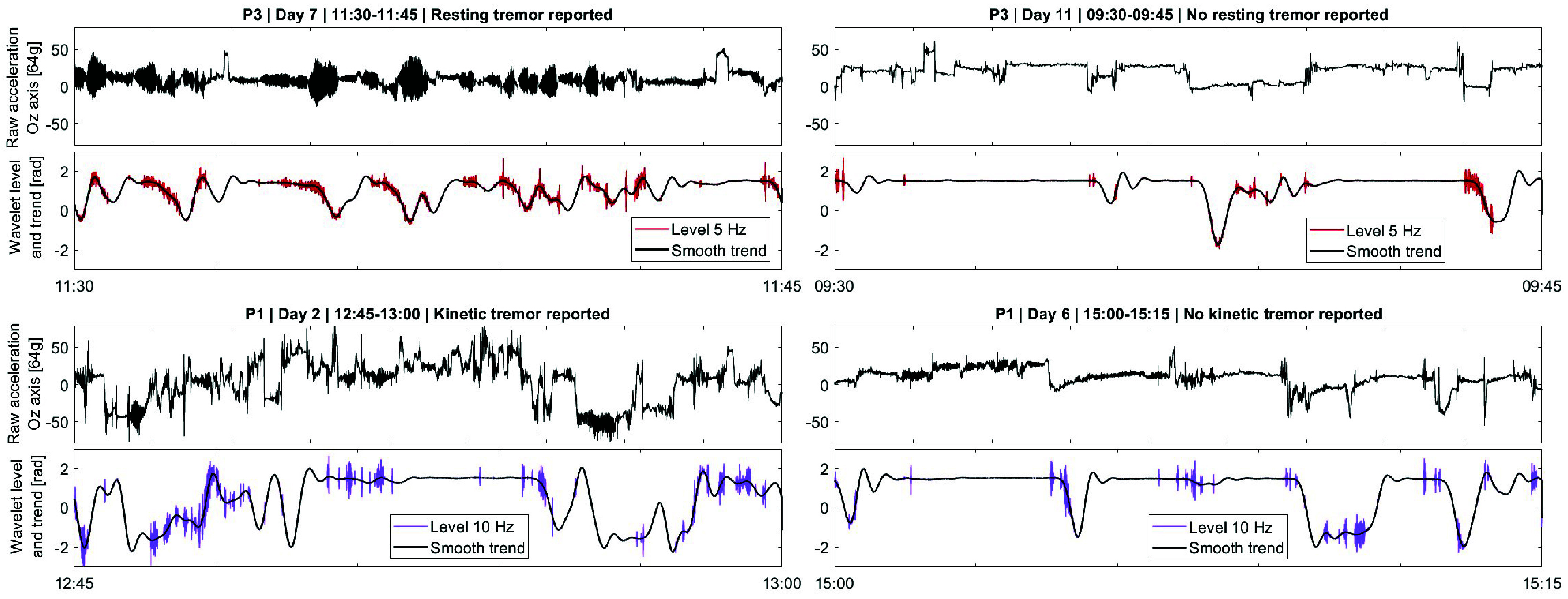
Comparative exemplification of disambiguation for resting (participants P3) and kinetic (participants P1) tremor in the most tremor-affected hand, during the 15-minute window, based on self-reports. (Larger version in Supplementary material.)

A comparative exemplification of RT vs. AT at 5 Hz vs. 10 Hz is shown in Fig. 5, in the most tremor-affected hand. The 15-minute windows are selected based on the self-reports of the two participants. Participant P3 reported RT on Day 7, but not on Day 11 while performing desk work on both days. Similarly, participant P1 reported AT on Day 2, but not on Day 6 while gardening on both days.

**FIGURE 6.**
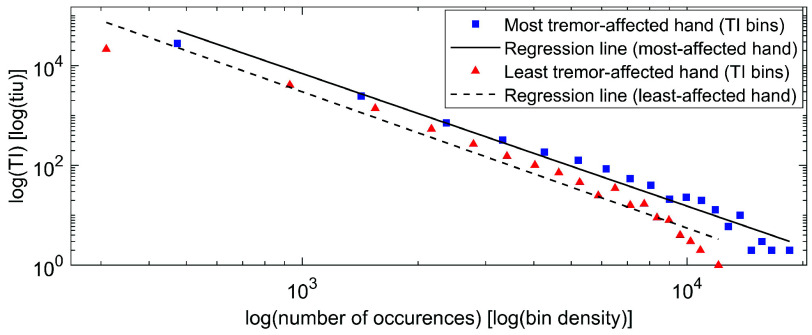
Results from the regression analysis of binned TI scores across all participants and all frequencies (log-log transformation).

Fig. 6 illustrates the result of the linear regression in the log-log domain for the PDFs of the TIs ( $\lambda = 20$ bins) for all participants and all frequencies, while Table 2 presents the characteristics of the TI distributions (kurtosis, skewness) and linearizations (slope, intercept), across all participants for each frequency as well as all together. The Supplementary material includes these results for each participant. Results shows higher kurtosis and skewness for the most tremor-affected hand, ranging from 1.5 (4Hz and 7-12Hz) to 3 times larger (5-6 Hz). At group level, the distributions of TIs are long-tailed for both hands. At each level, the intercepts show that this participant sample exhibits larger cumulative amplitudes in the oscillations of the higher-frequency movements.

**TABLE 2:** Kurtosis and Skewness (TI Distributions), Slope and Intercept (TI PDFs), for All Participants

Frequency	Kurtosis		Skewness		Slope		Intercept	
	T	nT	T	nT	T	nT	T	nT
All	56.04	32.93	6.04	4.42	−2.65	−2.73	27.19	26.86
3Hz	22.70	23.05	3.62	3.54	−2.17	−2.18	20.14	20.50
4Hz	65.82	25.02	6.28	3.91	−2.34	−2.19	22.12	20.96
5Hz	103.76	30.24	8.31	4.40	−2.27	−2.14	20.55	19.43
6Hz	102.33	30.62	8.28	4.45	−2.25	−2.10	20.80	19.37
7Hz	58.25	29.14	6.08	4.32	−2.05	−2.19	19.75	20.23
8Hz	35.84	26.87	5.03	4.13	−2.04	−2.12	19.95	19.84
9Hz	41.48	24.33	5.35	3.91	−2.21	−2.17	20.52	18.85
10Hz	43.56	22.67	5.45	3.76	−2.11	−2.18	20.31	19.14
11Hz	42.92	21.74	5.40	3.66	−2.12	−2.22	20.76	19.76
12Hz	41.51	20.97	5.30	3.58	−2.11	−2.22	20.96	19.90

T = most tremor-affected hand; nT = least tremor-affected hand.

The individual variation across participants is reflected by the correlation analysis between symptom load *L* (from clinical scores) and the number of separated TI frequencies *C* (Table 1). The Pearson coefficient $r = 0.88$ ( $p< 0.01$) shows a significant correlation between *L* and *C*. For the $L-C$ differences, the mean (−0.97), 95% CI $= [-2.59~~0.64]$, and paired samples t-test ( $p=1$) do not reject the null hypothesis, confirming the correlation analysis.

## IV. Discussion

In this article we designed a wavelet-based tremor quantification algorithm that is able to disambiguate tremor across the known tremor frequency ranges in free-living conditions. Quantifying tremor using wearable-derived signals gives us a more sensitive and objective measure, compared to clinical scales like MDS-UPDRS. This measure has potential to be used in clinical research, including treatment trials, but also, potentially, for routine monitoring in clinical practice, provided it is adequately and independently validated in larger cohorts. And can potentially alleviate the burden of keeping track of and reporting severity and occurrence of tremor.

In general, across participants, we observed a difference in TI distributions between the most tremor-affected hand and the least tremor-affected hand. This indicates that we can separate the tremor-dominant sides based on distribution features. The correlation analysis demonstrates a relationship with the clinical assessment: the types of tremor and their intensities are reflected by inter-hand separation for frequency ranges of corresponding breadth.

Individually, there is variation among participants in which frequencies show the greatest separation, as seen in the properties of the TI distributions (included in the Supplementary material). Given the symptom heterogeneity of PD [39], [40] and of PD tremor in particular [17], [18], [19], this is expected.

In Mode I, the TI quantifies tremor over a wide range of frequencies (3-12 Hz) associated with PD tremor and tremor in general [4], allowing to map where a specific person typically exhibits tremor. This includes the common range of 4-6 Hz for RT and re-emergent postural tremor [41], and covers the higher frequency range of postural and kinetic PD tremor [42], [43], [44]. As higher kurtosis has been previously linked to PD tremor using electromyography signals [45], [46], specifically to increasing severity investigated via acceleration signals [38], the high TI kurtosis at 4-6 Hz may thus reflect rest-tremor and re-emergent postural tremor in PD, which echos the clinical MDS-UPDRS part III assessment across our participant group.

At higher frequencies, which are often associated with AT [42], [43], [44] and its harmonics [47], the TI allows for symptom tracking in free-living settings, where unconstrained daily activities affect the way tremor is exhibited. These factors include stress [5], arm positioning [48], and weight loading [47], which may shift the frequency from the typical AT range.

When PD tremor is measured in controlled conditions, clear frequency peaks are typically found [5], [35], [49], [50]. Longer measurements give a better mean, reflective of patients’ tremor over time, as opposed to a single session snapshot, which has revealed peak inconsistencies over time [51], and smaller multiple-peaks in the case of lower symptom severity [52]. Our analysis shows similar findings for the study participants. The dynamic variability in tremor frequency and amplitude is a result of physiological variation, activity, and dopaminergic medication intake [53], [54], [55], all of which the participants would have experienced over the two weeks of data collection.

As RT and re-emergent postural tremor can potentially propagate from lower to higher frequencies [47], the TI offers an unique opportunity to further investigate this phenomenon. Based on self-reported diaries [25] from the current participants, we were able to empirically observe the effects of daily living on the different wavelet levels (Fig. 5). A more systematic analysis, however, requires detailed and objective logging of activities, which is challenging, as video recordings would raise issues of dignity and privacy in free-living settings.

At the same time, while the multi-resolution analysis extracts levels at specific frequencies, the Daubechies wavelet decomposition allows for bleed-through from in-between the integer levels [56]. Thus, the TI is able to capture and preserve smaller peaks around the main frequencies where information on tremor might be hiding. As long as the raw signal sampling frequency permits it and resampling is reliable, our proposed algorithm can accommodate other frequency sets, which would help future efforts at more-precise identification of the RT and AT specific ranges and their outliers.

In Mode II, when measured over longer periods of time, TI patterns relating to fluctuations and progression of disease may emerge. This is important for the relationship between tremor and other clinical features of PD. Tremor is reported to be a particularly bothersome symptom [57], [58], with a higher likelihood for people with PD to report low quality of life [59]. The TI can potentially elucidate how tremor in particular evolves, as currently it is known as more independent of other clinical characteristics and PD dysfunction [60], [61]. The TI can also increase the precision of treatment assessment, as tremor in particular shows variation in responses to levodopa [62]: dopamine-responsive, intermediate, or resistant [40]. By mapping tremor over time, the TI can equip clinicians with objective insights complementary to the standard clinical examination and uncover treatment-resistant tremor types.

The strength of our proposed tremor quantification method lies in its simplicity. By focusing on energy within known tremor frequency bands, our algorithm is more translatable to clinical use. We envision that a refined TI will provide sensitive outcome measures for treatment studies in PD research, and thus potentially uncover disease modifying therapies that are not detectable with standard outcome measures such as the MDS-UPDRS III. For patients, this measure would unburden them by passively monitoring their symptom occurrence and the trajectory of the disease, reducing the labor of self-reporting. For attending clinicians, clinically relevant summaries from objective, continuous, long-term monitoring would improve decision-making.

Real-world implementation brings, however, specific challenges. Thus, ensuring that wearables are fitted properly is critical when recording in unsupervised free-living conditions, as loosely-latched sensors may introduce measurement noise or fail to record altogether. In the current study, we evaluated that the Empatica E4 devices were adequately fitted based on: 1. the data collection protocol involved visiting participants every 48-hours, when the researcher would help mount the device and make sure that participants could properly re-fit it on the wrist if needed; 2. Empatica E4 stops recording or presents obvious error artifacts when not properly worn, which helps prune the signals. Our preliminary study evaluating these devices in the same participants indicated consistent sampling (e.g., high uptime) and data plausibility (see Table 6 in [25]). Improper mounting remains a risk in unsupervised conditions, and for Empatica E4 the short battery life could become burdensome; at the same time, improved wearable designs continue to address some of these physical device challenges.

### A. Limitations

Our sample size was $N = 7$ participants, thus the results of the correlation analysis must be taken in this context. The International Parkinson and Movement Disorder Society’s commissioned roadmap [63] for patient-centered implementation of technologies in care and research reflects on the expectations for new digital outcome measures to correlate with current clinical assessment scales. They surmise that discrepancies between new and “gold standard” measures are desirable for complementarity. Nevertheless, new measures require clinical validation, for which we consider the current results an important and promising step forward.

The study had a wide variation in terms of available 24-hours segments per handedness side, for each participant. The missing data was caused mainly by device malfunctions, when sudden stops in recording occurred. In the precursor article [25], we dive deeper into the practicalities of Empatica E4, where we identified human error as partly responsible for missing data. For instance, the event marker button doubles as power/restart, which led to the device being turned off for some participants when attempting to mark events. In this article we chose a very conservative threshold and removed entire days instead of smaller segments, for inter-day consistency.

The accelerometer data was collected on both upper limbs, but asynchronously, which may have impacted the analysis due to fluctuation of symptoms and daily activities, the latter being also dependent on handedness. Including TI calculations for the nighttime data may have affected the breadth of the inter-hand separation due to the addition of mostly low TI values, thus shifting the distributions. Furthermore, we reduced the 3-axis accelerometry to a single dimension in an attempt to broadly mitigate interference from daily activities, which ultimately excludes some components of tremor. We made this choice by balancing the useful tremor information against possible loss; for instance, the $Ox$ axis measures would affect TI amplitude, but not its relative evolution over time. Better precision would be possible with more degrees of freedom for the kinematic analysis, especially rotation via 3-axis gyroscope and gravitation orientation via magnetometer.

### B. Future Work

In its current form, the design of the proposed TI accounts, to a certain extent, for the potential effects of tremor types, harmonics, and factors of daily living in free-living conditions. Even in the presence of possible artefacts from non-tremor movements, our findings show that the TI has the potential to provide clinicians and researchers with more information, which can ultimately inform the assessment of treatment effects and the management of symptoms. Meanwhile, the weaknesses we identified will make a solid basis for improving this outcome measure. Our next steps involve testing and validating the proposed TI in a larger cohort, with synchronous data collection on both sides. We plan to add a gyroscope and, if possible, a magnetometer for multi-modal sensing. We will utilize the current results to refine the calculation of the TI toward more precise quantification for different tremor types. We will implement a process of co-design with the involvement of our neurologist co-authors and users with PD. Ultimately, we envision the deployment of the proposed metric in real-world clinical practice and research.

## V. Conclusion

We designed an algorithm for tremor quantification and disambiguated tremor-related movement at relevant frequencies. Comparing within subjects, we show a separation of the tremor-affected sides while considering the heterogeneity of PD and the unique daily routines of the individual. We show that a context-independent metric of energy across multiple known tremor frequencies may be a viable method of quantifying tremor in free-living conditions, improving the outcome measures for PD research and adding value to the clinical assessment of treatment response and disease development.

## Supplementary Materials

Supplementary Materials
