# Supplementary material for: Wavelet-Based Tremor Quantification From Wrist-Worn Sensor Data in Home-Dwelling People With Parkinson’s Disease
Source: IEEE J Transl Eng Health Med. 2025 Dec 25;14:19–28. doi: 10.1109/JTEHM.2025.3648704 (PMC12885486; doi:10.1109/JTEHM.2025.3648704)
Supplement: Supplementary Materials [file supp1-3648704.pdf]

Supplementary Material

Cumulative distributions across all participants

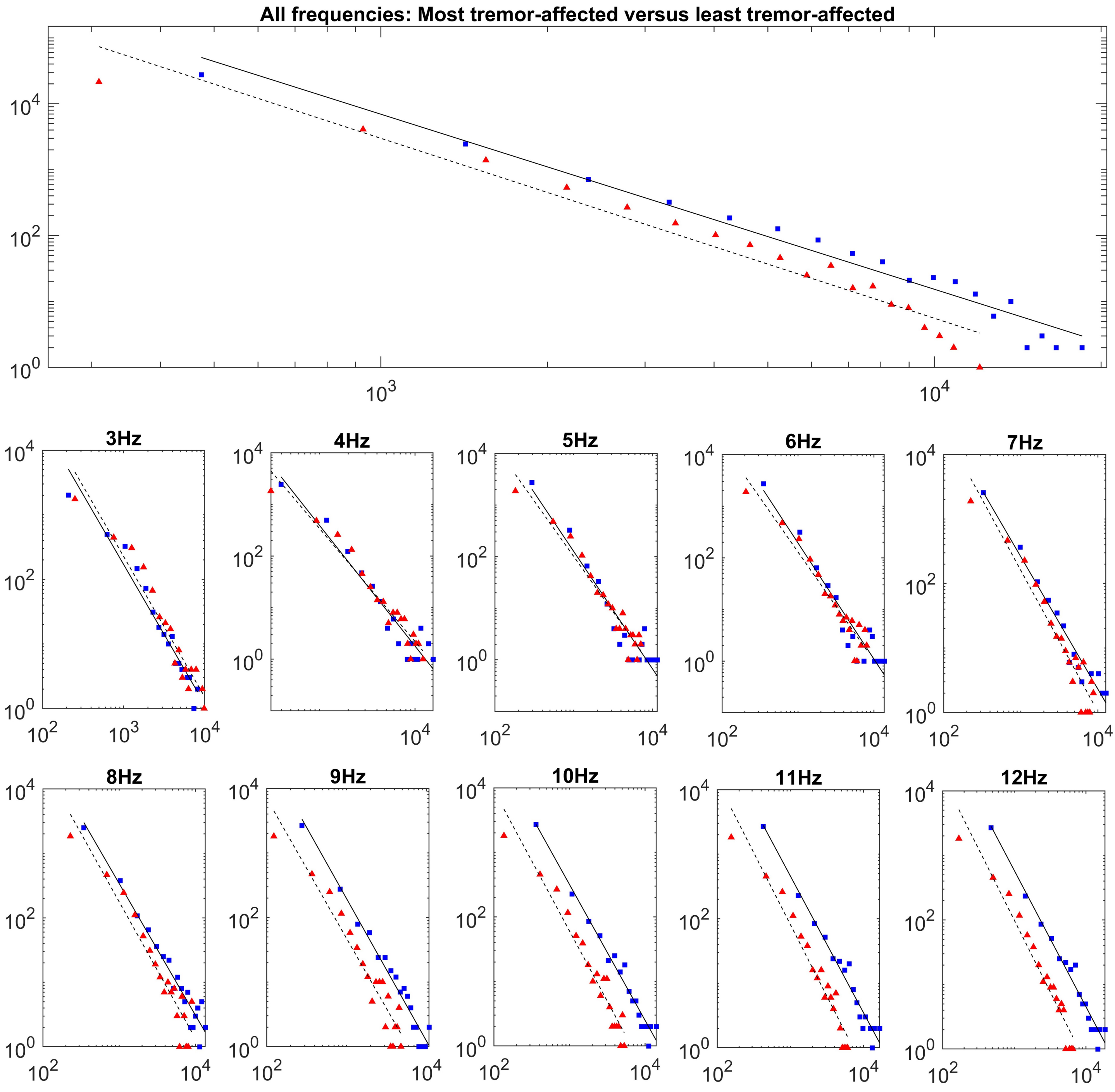

Figure 1: Results from the regression analysis of binned TI scores across all participants and all days on data from the most tremor-affected hand (blue, squares) and least tremor-affected hand (red, triangles). Top-bottom rows: (1) log-log transformation of all frequencies, (2-3) log-log transformation of each frequency (3Hz to 12Hz). Hz = Hertz. Vertical axes: log(TI) [log(tiu)]. Horizontal axes: log(number of occurrences) [log(bin density)].

Individualized distributions for all frequencies

This section contains results from the regression analysis of binned TI scores from all days for each individual participant. Each figure shows data from the most tremor-affected hand (blue, squares) and least tremor-affected hand (red, triangles). Each plot is a log-log transformation of a specific frequency (3Hz to 12Hz). Hz = Hertz. Horizontal axes: log(number of occurrences) [log(bin density)], and vertical axes: log(TI) [log(tiu)]. Each figure has the participant ID in the title of each plot, followed by the specific frequency. Participants are labeled as 'P' followed by an ID number (e.g., P1, P2, etc.). For each participant, the associated table shows the characteristics of the distributions for all frequencies as well as for each frequency.

Participant P1

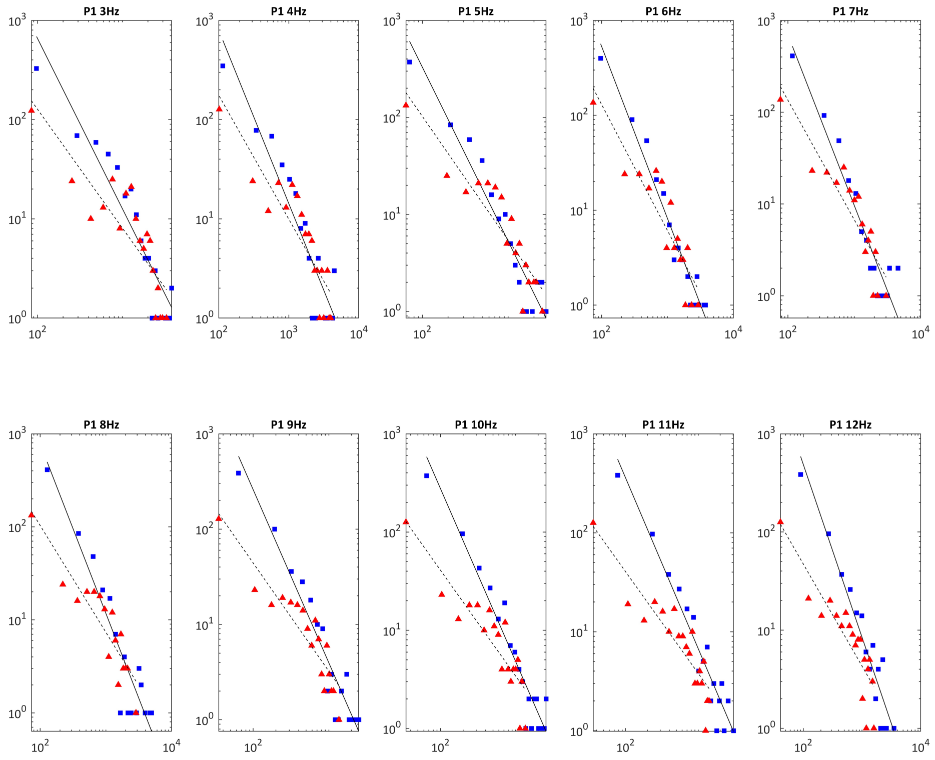

Figure 2: Log–log regression of binned TI scores (3–12 Hz) for most tremor-affected hand (blue squares) and least affected hand (red triangles). Horizontal: log(occurrences) [log(bin density)], Vertical: log(TI) [log(tiu)]. P1 = Participant 1

Frequency	Kurtosis		Skewness		Slope		Intercept	
	T	NT	T	NT	T	NT	T	NT
3Hz	11.63	3.74	2.46	1.14	-1.72	-1.20	14.39	10.36
4Hz	17.97	5.12	3.23	1.48	-1.75	-1.25	14.73	10.92
5Hz	21.40	5.99	3.67	1.64	-1.79	-1.28	14.06	10.51
6Hz	27.20	6.15	4.22	1.65	-1.82	-1.33	14.65	11.00
7Hz	31.48	5.65	4.57	1.57	-1.86	-1.30	15.13	10.91
8Hz	28.36	4.75	4.35	1.43	-1.82	-1.15	15.04	9.92
9Hz	22.40	4.15	3.86	1.33	-1.82	-1.14	13.92	9.01
10Hz	18.28	3.93	3.47	1.29	-1.76	-1.08	13.72	8.66
11Hz	16.25	3.88	3.25	1.28	-1.73	-1.03	13.85	8.45
12Hz	15.45	3.89	3.14	1.29	-1.76	-1.04	14.30	8.64

Table 1: Kurtosis and skewness of most tremor affected hand (T) and least tremor-affected hand (NT) distributions. Slope and intercept of regression analysis of binned log-log transformed data for T and NT.

Participant P2

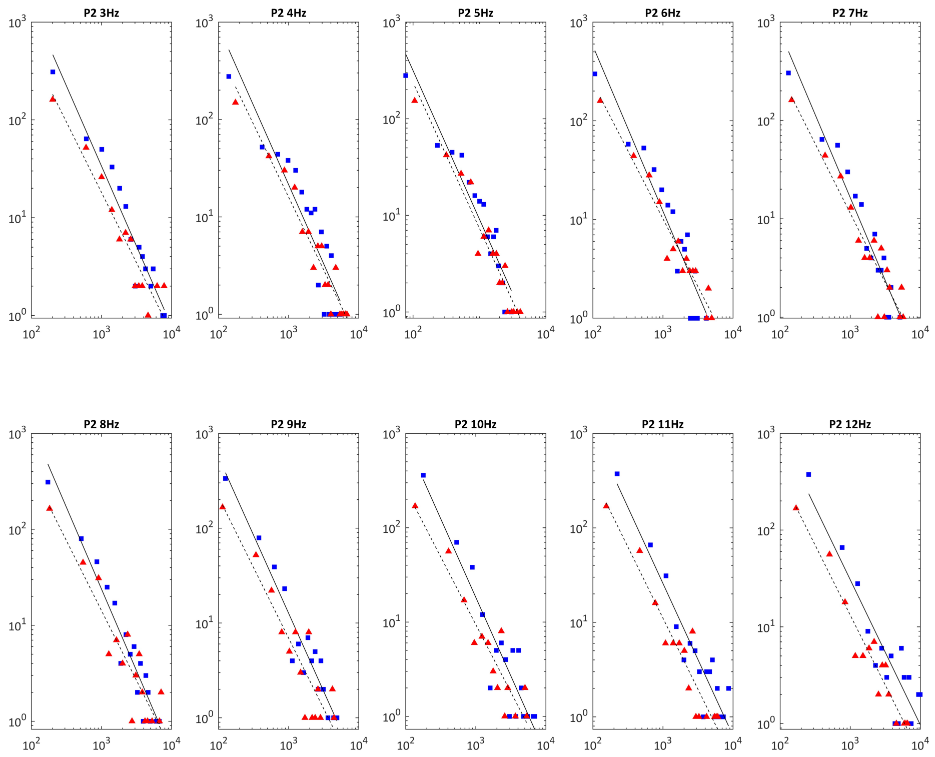

Figure 3: Log–log regression of binned TI scores (3–12 Hz) for most tremor-affected hand (blue squares) and least affected hand (red triangles). Horizontal: log(occurrences) [log(bin density)], Vertical: log(TI) [log(tiu)]. P2 = Participant 2

Frequency	Kurtosis		Skewness		Slope		Intercept	
	T	NT	T	NT	T	NT	T	NT
3Hz	15.95	20.75	3.07	3.69	-1.64	-1.44	14.83	12.83
4Hz	8.17	12.07	2.12	2.80	-1.62	-1.49	14.28	13.08
5Hz	7.87	12.03	2.08	2.74	-1.54	-1.51	12.83	12.42
6Hz	10.86	13.11	2.49	2.91	-1.67	-1.38	14.06	11.83
7Hz	12.74	13.89	2.75	3.04	-1.69	-1.41	14.45	12.19
8Hz	15.04	15.64	3.09	3.22	-1.70	-1.46	14.93	12.72
9Hz	17.86	17.06	3.50	3.37	-1.65	-1.49	13.90	12.20
10Hz	19.66	17.75	3.77	3.44	-1.67	-1.43	14.41	12.14
11Hz	20.88	17.64	3.94	3.42	-1.61	-1.51	14.38	12.81
12Hz	22.13	16.66	4.07	3.33	-1.50	-1.45	13.79	12.56

Table 2: Kurtosis and skewness of most tremor affected hand (T) and least tremor-affected hand (NT) distributions. Slope and intercept of regression analysis of binned log-log transformed data for T and NT.

Participant P3

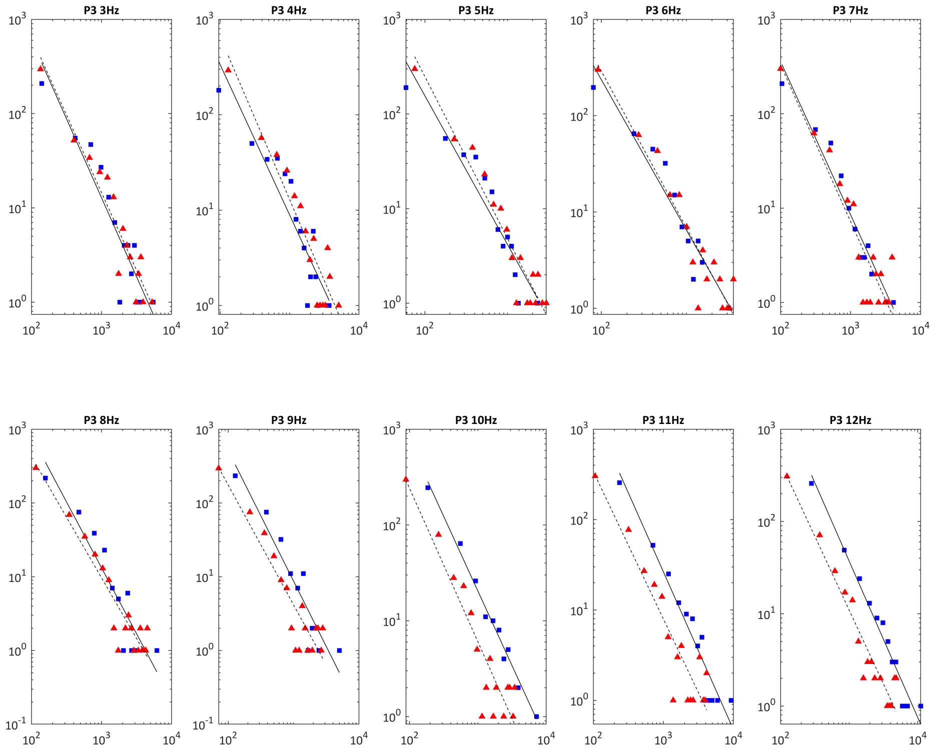

Figure 4: Log–log regression of binned TI scores (3–12 Hz) for most tremor-affected hand (blue squares) and least affected hand (red triangles). Horizontal: log(occurrences) [log(bin density)], Vertical: log(TI) [log(tiu)]. P3 = Participant 3

Frequency	Kurtosis		Skewness		Slope		Intercept	
	T	NT	T	NT	T	NT	T	NT
3Hz	16.10	14.38	2.95	2.92	-1.68	-1.65	14.13	14.06
4Hz	10.23	15.33	2.28	3.09	-1.58	-1.70	13.08	14.34
5Hz	12.11	19.32	2.31	3.56	-1.57	-1.71	12.29	13.40
6Hz	18.50	21.65	2.93	3.87	-1.56	-1.63	12.67	13.17
7Hz	23.84	21.07	3.42	3.88	-1.63	-1.66	13.37	13.45
8Hz	32.97	21.18	4.04	3.91	-1.79	-1.64	14.93	13.62
9Hz	34.14	22.75	4.19	4.06	-1.77	-1.63	14.39	12.65
10Hz	29.13	24.00	3.98	4.17	-1.56	-1.60	13.78	12.86
11Hz	25.59	24.60	3.81	4.22	-1.75	-1.63	15.34	13.34
12Hz	23.96	25.23	3.74	4.27	-1.70	-1.60	15.34	13.45

Table 3: Kurtosis and skewness of most tremor affected hand (T) and least tremor-affected hand (NT) distributions. Slope and intercept of regression analysis of binned log-log transformed data for T and NT.

Participant P4

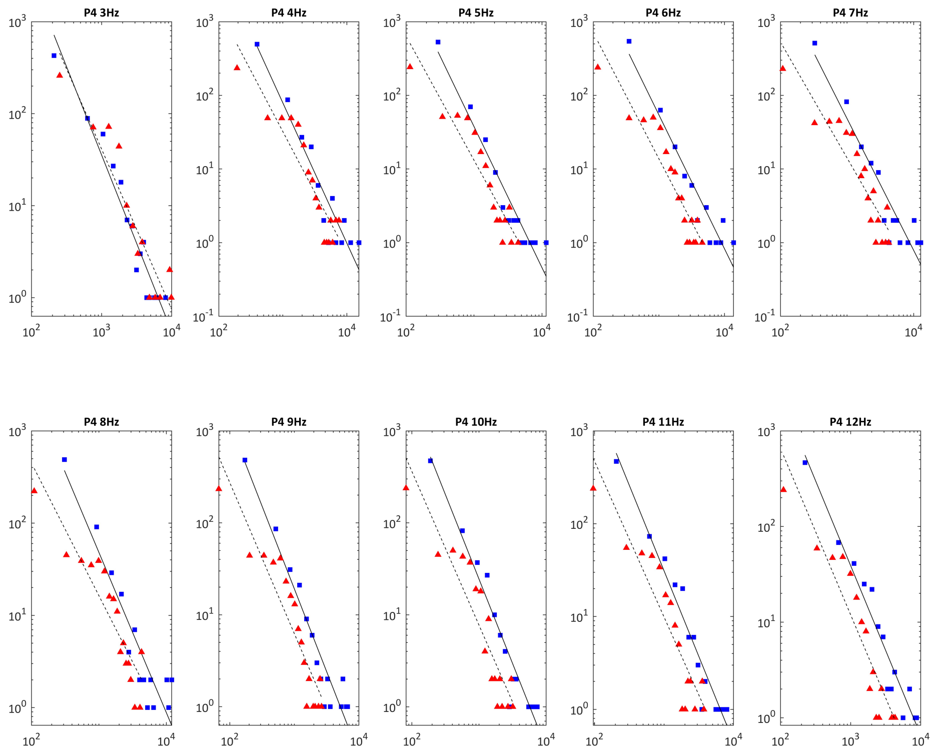

Figure 5: Log–log regression of binned TI scores (3–12 Hz) for most tremor-affected hand (blue squares) and least affected hand (red triangles). Horizontal: log(occurrences) [log(bin density)], Vertical: log(TI) [log(tiu)]. P4 = Participant 4

Frequency	Kurtosis		Skewness		Slope		Intercept	
	T	NT	T	NT	T	NT	T	NT
3Hz	24.03	28.47	3.72	4.05	-1.92	-1.75	16.85	15.78
4Hz	48.20	13.12	5.54	2.63	-1.91	-1.66	17.54	14.91
5Hz	62.51	11.55	6.53	2.40	-1.92	-1.69	16.87	14.23
6Hz	62.58	10.68	6.68	2.34	-1.82	-1.71	16.57	14.46
7Hz	52.49	9.20	6.19	2.14	-1.79	-1.58	16.23	13.53
8Hz	46.01	8.67	5.78	2.04	-1.74	-1.44	15.88	12.73
9Hz	42.84	9.73	5.50	2.15	-1.80	-1.64	15.39	13.18
10Hz	39.09	11.38	5.13	2.35	-1.84	-1.67	15.87	13.59
11Hz	34.08	12.91	4.67	2.51	-1.84	-1.68	16.17	13.89
12Hz	29.30	14.40	4.26	2.65	-1.78	-1.74	15.94	14.51

Table 4: Kurtosis and skewness of most tremor affected hand (T) and least tremor-affected hand (NT) distributions. Slope and intercept of regression analysis of binned log-log transformed data for T and NT.

Participant P5

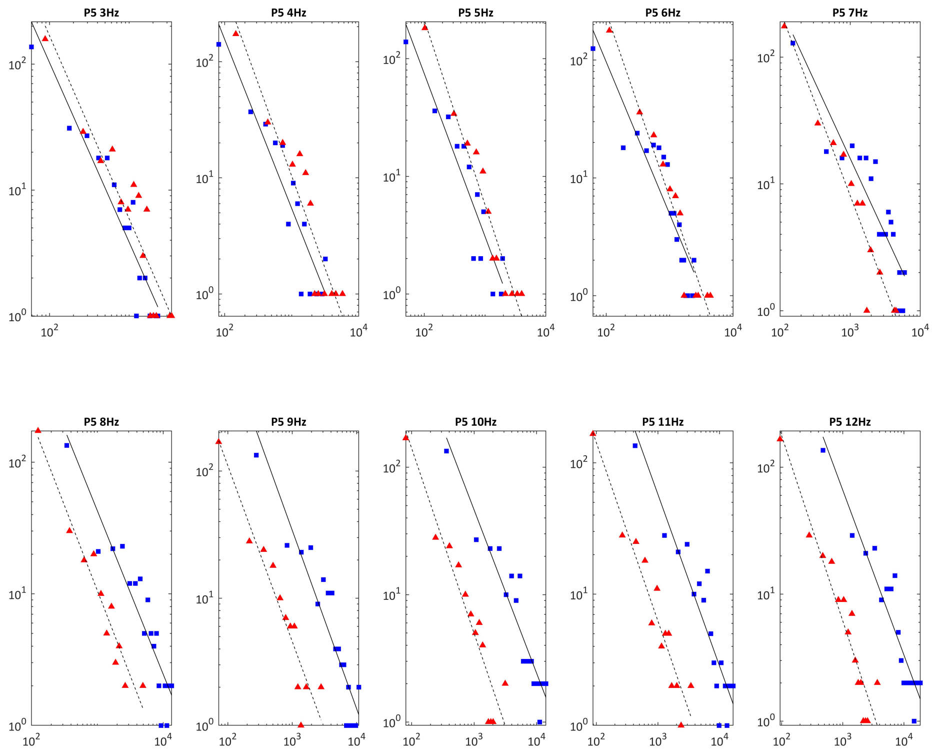

Figure 6: Log–log regression of binned TI scores (3–12 Hz) for most tremor-affected hand (blue squares) and least affected hand (red triangles). Horizontal: log(occurrences) [log(bin density)], Vertical: log(TI) [log(tiu)]. P5 = Participant 5

Frequency	Kurtosis		Skewness		Slope		Intercept	
	T	NT	T	NT	T	NT	T	NT
3Hz	8.65	9.78	2.12	2.24	-1.42	-1.45	11.19	11.80
4Hz	14.65	19.50	2.87	3.33	-1.46	-1.59	11.76	13.35
5Hz	14.64	27.12	2.83	4.10	-1.39	-1.57	10.70	12.62
6Hz	6.05	25.44	1.61	3.96	-1.29	-1.57	10.53	12.78
7Hz	5.02	21.20	1.52	3.52	-1.20	-1.46	11.04	12.18
8Hz	6.31	19.16	1.77	3.30	-1.24	-1.29	12.34	11.31
9Hz	7.05	18.04	1.90	3.20	-1.40	-1.38	13.21	11.06
10Hz	7.30	16.69	1.94	3.10	-1.28	-1.45	12.68	11.64
11Hz	7.43	15.16	1.96	2.96	-1.31	-1.36	13.08	11.22
12Hz	7.61	13.67	1.99	2.81	-1.30	-1.46	13.16	11.89

Table 5: Kurtosis and skewness of most tremor affected hand (T) and least tremor-affected hand (NT) distributions. Slope and intercept of regression analysis of binned log-log transformed data for T and NT.

Participant P6

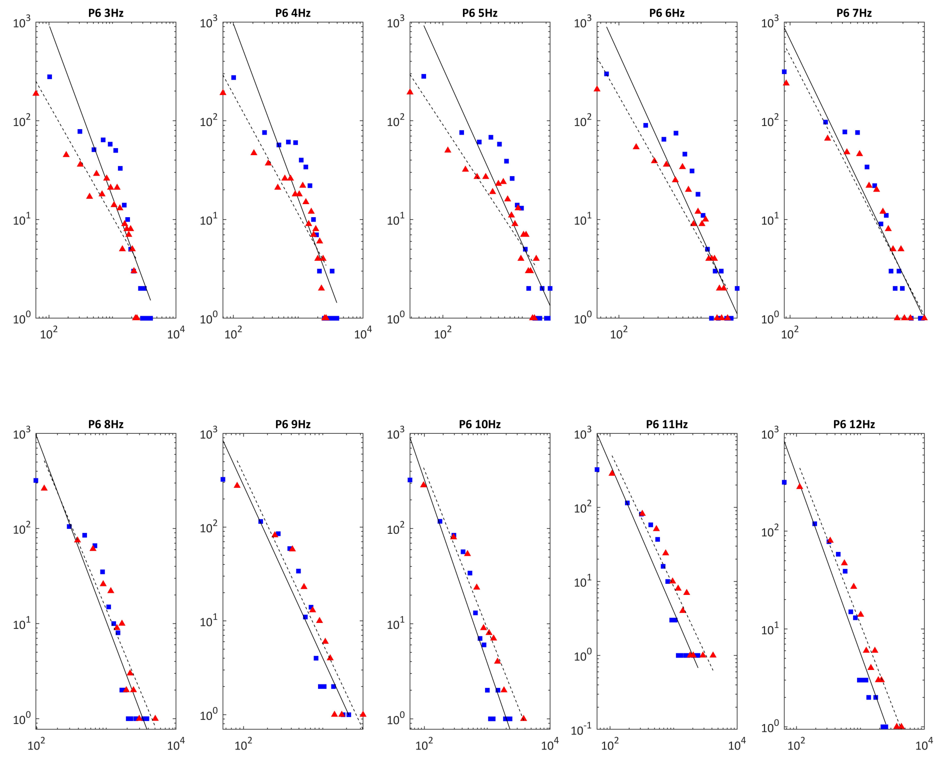

Figure 7: Log–log regression of binned TI scores (3–12 Hz) for most tremor-affected hand (blue squares) and least affected hand (red triangles). Horizontal: log(occurrences) [log(bin density)], Vertical: log(TI) [log(tiu)]. P6 = Participant 6

Frequency	Kurtosis		Skewness		Slope		Intercept	
	T	NT	T	NT	T	NT	T	NT
3Hz	8.28	3.40	1.79	1.14	-1.74	-1.14	14.88	10.22
4Hz	8.13	3.70	1.80	1.21	-1.77	-1.21	15.03	10.81
5Hz	9.73	4.25	2.04	1.34	-1.78	-1.23	14.05	10.16
6Hz	11.83	5.70	2.30	1.62	-1.83	-1.48	14.58	11.97
7Hz	14.28	10.41	2.51	2.17	-1.83	-1.70	14.91	13.95
8Hz	16.61	18.71	2.70	2.87	-1.95	-1.76	15.81	14.79
9Hz	18.26	25.69	2.84	3.40	-1.84	-1.80	14.10	14.20
10Hz	18.73	27.48	2.90	3.59	-1.93	-1.67	14.67	13.72
11Hz	18.03	25.75	2.87	3.59	-1.99	-1.82	15.11	14.74
12Hz	16.61	23.57	2.76	3.54	-1.82	-1.68	14.29	14.03

Table 6: Kurtosis and skewness of most tremor affected hand (T) and least tremor-affected hand (NT) distributions. Slope and intercept of regression analysis of binned log-log transformed data for T and NT.

Participant P7

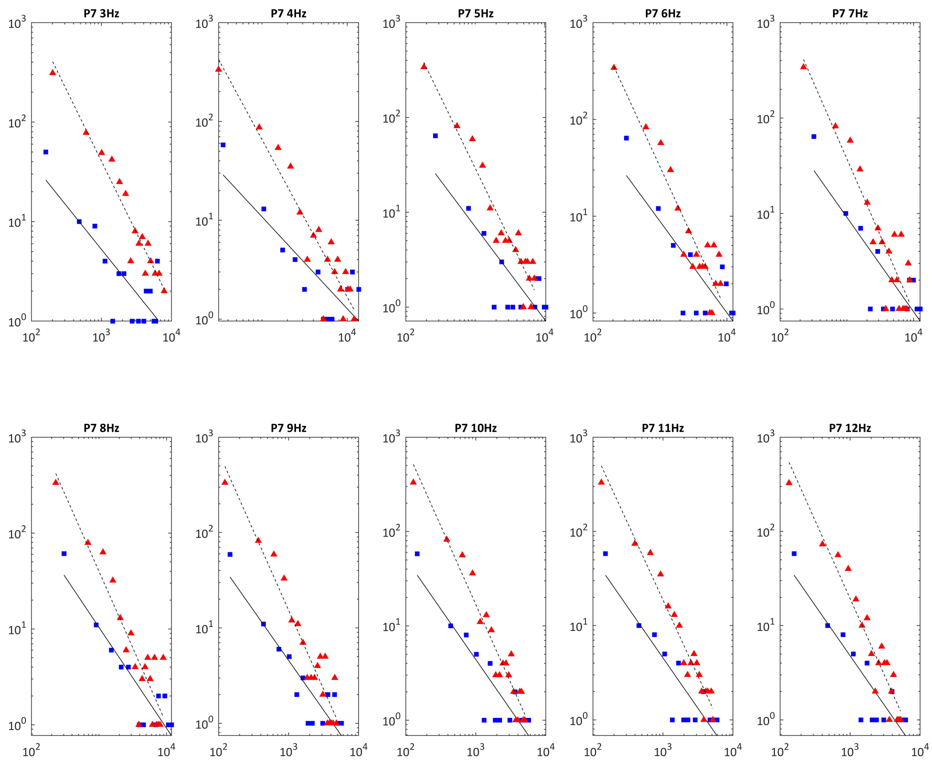

Figure 8: Log–log regression of binned TI scores (3–12 Hz) for most tremor-affected hand (blue squares) and least affected hand (red triangles). Horizontal: log(occurrences) [log(bin density)], Vertical: log(TI) [log(tiu)]. P7 = Participant 7

Frequency	Kurtosis		Skewness		Slope		Intercept	
	T	NT	T	NT	T	NT	T	NT
3Hz	5.11	11.03	1.83	2.59	-0.88	-1.44	7.73	13.62
4Hz	8.89	13.38	2.57	3.05	-0.93	-1.59	8.81	15.18
5Hz	10.43	13.90	2.85	3.18	-0.98	-1.51	8.70	13.80
6Hz	10.98	14.28	2.93	3.24	-0.95	-1.50	8.72	13.87
7Hz	11.14	14.50	2.93	3.24	-0.99	-1.60	9.06	14.70
8Hz	10.93	14.54	2.87	3.21	-1.05	-1.59	9.61	14.70
9Hz	10.64	14.16	2.79	3.13	-1.05	-1.66	8.74	14.19
10Hz	10.56	13.56	2.77	3.02	-1.07	-1.67	8.87	14.33
11Hz	10.66	12.88	2.77	2.91	-1.07	-1.62	8.91	14.11
12Hz	10.76	12.18	2.78	2.80	-1.07	-1.66	8.96	14.42

Table 7: Kurtosis and skewness of most tremor affected hand (T) and least tremor-affected hand (NT) distributions. Slope and intercept of regression analysis of binned log-log transformed data for T and NT.

Larger versions of figures 4 and 5

The figures depict the wavelet transform at scales of 2 minutes and 15 minutes, showing the disambiguation of the raw acceleration data, the 5 Hz and 10 Hz levels, and the smooth.

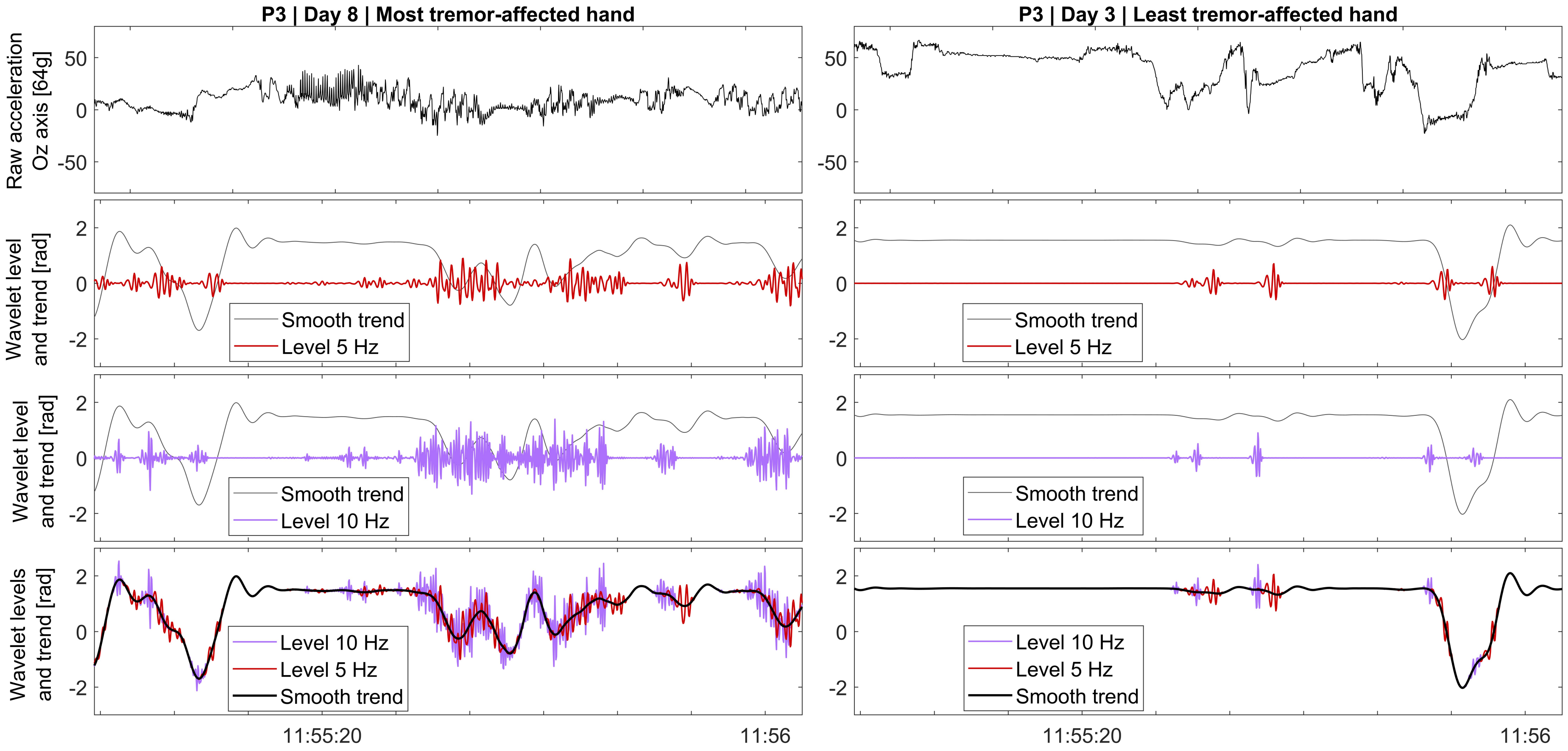

Figure 9: Exemplification of the 5 & 10 Hz wavelet levels with the smooth for participant P3 over 1 minute, while performing the same activity (desk work), with self-reported experienced tremor on Day 8.

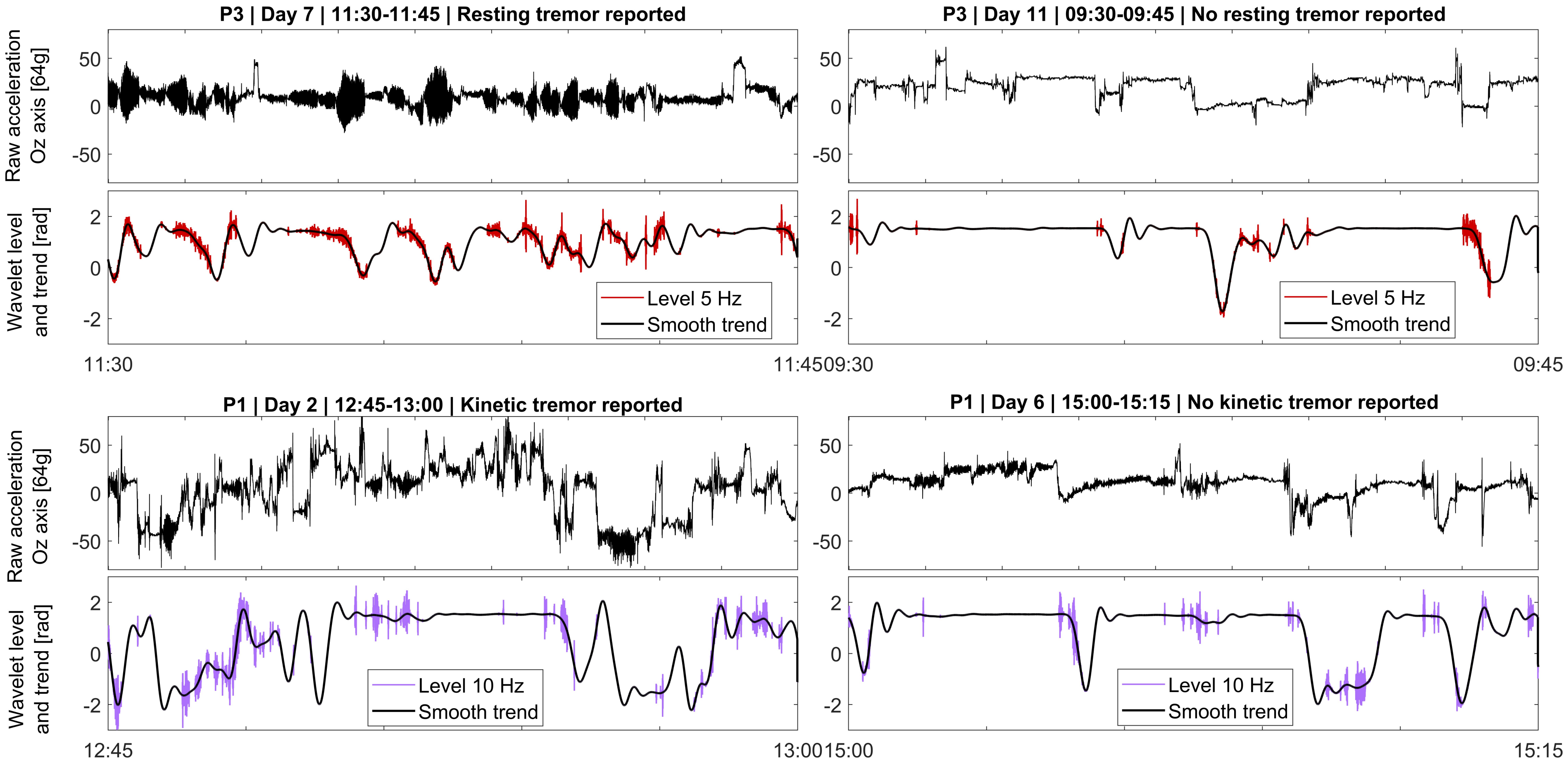

Figure 10: Comparative exemplification of disambiguation for resting (participants P3) and kinetic (participants P1) tremor in the most tremor-affected hand, during the 15-minute window, based on self-reports

Disambiguation examples

This section contains two figures exemplifying the transformation of acceleration signals to tremor index (TI) from study participant (P3) over 24 hours. The two figures contain Raw Oz axis acceleration, Daubechies wavelet levels disentangling specific frequencies (3Hz to 12Hz) and calculated TI over 15-minute sampling window intervals. Figure 11 shows the most tremor-affected hand and Figure 12 shows the least tremor-affected hand.

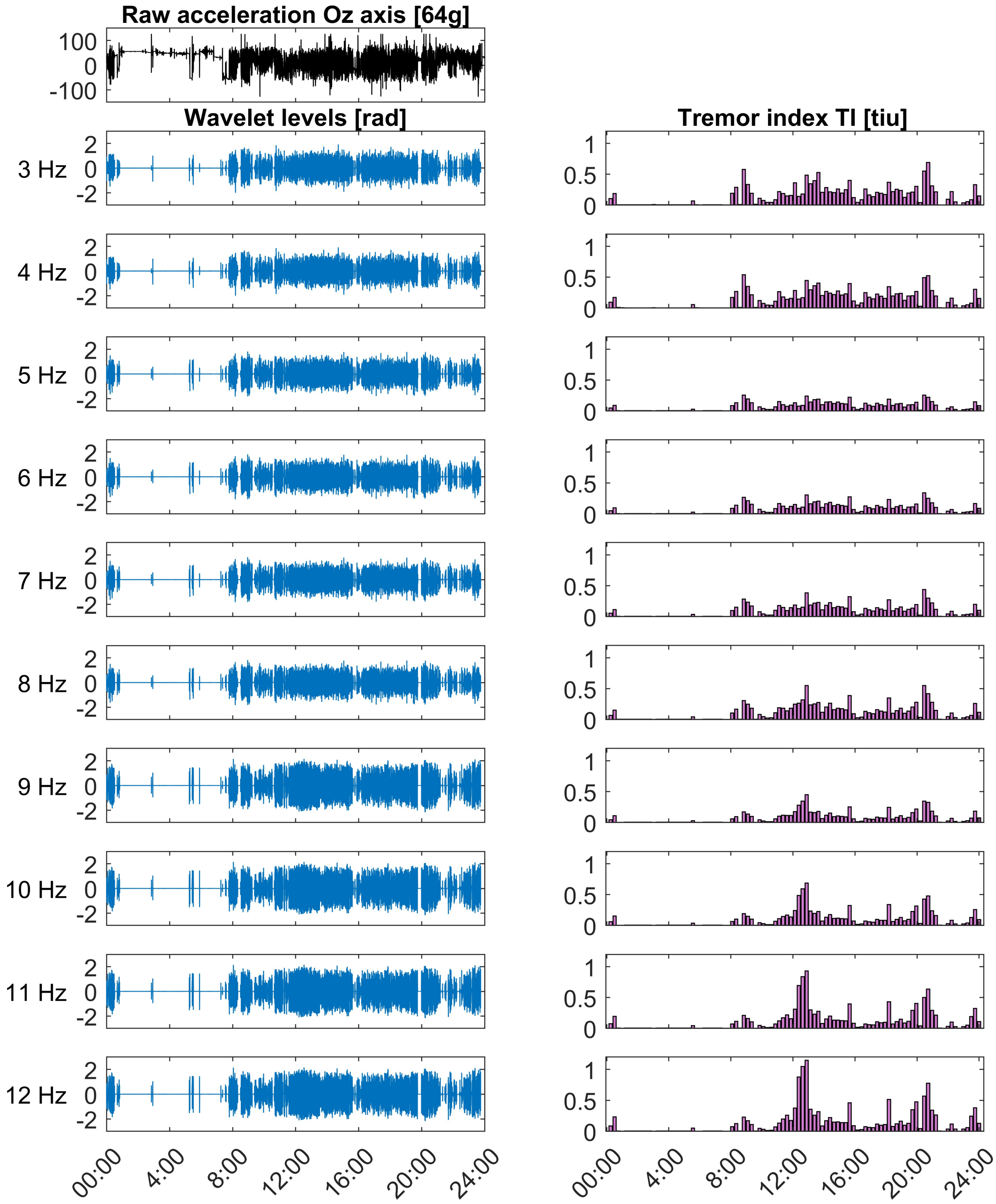

Figure 11: Study participant (P3) over 24 hours for **most tremor-affected side**. Left side consists of raw Oz-axis acceleration and Daubechies wavelet levels (3Hz to 12Hz); Right side consists of TI calculated over the 15-minute ST intervals. Tiu = tremor index unit. (64 units = 1g).

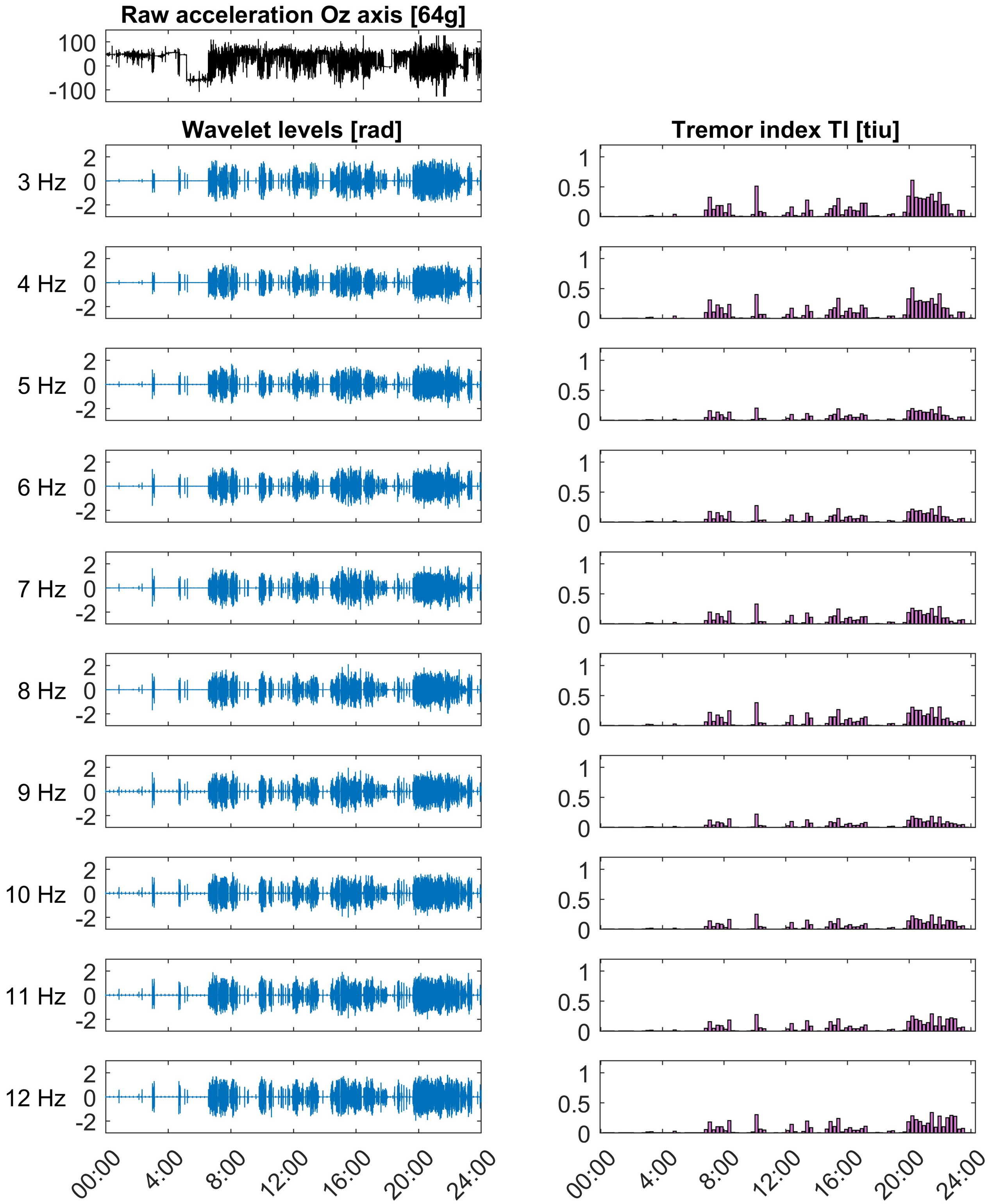

Figure 12: Study participant (P3) over 24 hours for **least tremor-affected side**. Left side consists of raw Oz-axis acceleration and Daubechies wavelet levels (3Hz to 12Hz); Right side consists of TI calculated over the 15-minute ST intervals. Tiu = tremor index unit. (64 units = 1g).
